# Facile Synthesis of MgO-Modified Carbon Adsorbents with Microwave- Assisted Methods: Effect of MgO Particles and Porosities on CO_2_ Capture

**DOI:** 10.1038/s41598-017-06091-5

**Published:** 2017-07-18

**Authors:** Young-Jung Heo, Soo-Jin Park

**Affiliations:** Department of Chemistry, Inha University, 100 Inharo, Incheon, 22212 Korea

## Abstract

In this study, magnesium oxide (MgO)-modified carbon adsorbents were fabricated using a nitrogen-enriched carbon precursor by microwave-assisted irradiation for CO_2_ capture. The X-ray diffraction (XRD) patterns showed the characteristic diffraction peaks of MgO at 43° and 62.5°, and no impurities were apparent. By changing the microwave reaction time, the spherical structure of the parent material was transformed to a hybrid structure with MgO crystalline particles in a carbon matrix. The morphology evolution and properties of the prepared materials were also investigated using transmission electron microscopy and N_2_ adsorption, respectively. On optimising the conditions, the prepared sample attained a high CO_2_ uptake of 1.22 mmol/g (5.3 wt.%) under flue gas conditions (15% CO_2_ in N_2_). It was found that MgO affected the CO_2_ capture behaviour by enhancing the fundamental characteristics of the carbon surfaces.

## Introduction

Global warming is a continuing concern for human society. The main anthropogenic greenhouse gas is carbon dioxide (CO_2_), which is released primarily from the combustion of fossil fuels used in power generation and manufacturing industries^1–5^. The International Energy Agency (IEA) predicts that fossil fuels will remain the dominant energy source until the year 2030^6^. CO_2_ capture, storage and utilization have therefore become a target for materials research communities worldwide. CO_2_ capture and utilization in post-combustion steps are currently considered the most practical option for the reduction of greenhouse gases^7, 8^. There are many CO_2_ capture methods, such as solvent-based chemisorption, solid adsorbent-based physisorption, carbonate looping and the oxy-fuel process^9–12^. An efficient sorbent has several prominent characteristics: large CO_2_ adsorption uptakes, fast adsorption and desorption kinetics, favourable adsorption and desorption temperature ranges and excellent cycling stability. Aqueous amine solutions are currently the most common carbon capture adsorbents with these properties, and they are used for the removal of CO_2_ from industrial gas emissions. However, amines can become problematic during operating and regeneration processes^9, 13^. There is now significant interest in the development of solid carbon adsorbents that can selectively adsorb gases such as CO_2_, H_2_ and CH_4_^14–18^. Because of their large surface areas, porosities, abundance and cost efficiency, carbon materials are often ideal adsorbents^19–21^. Furthermore, such materials have advantages in terms of ease of handling, pore structures, surface characteristics and low regeneration energies^22, 23^. Because of these characteristics, changing the surface structures and weight percentages of various embedded elements affects the properties of these materials, enhancing their selectivities and capture and storage abilities. Carbon materials have therefore already been considered as attractive candidate sorbents for CO_2_ capture in the development of alternative clean and sustainable energy technologies.

**Figure 1:**
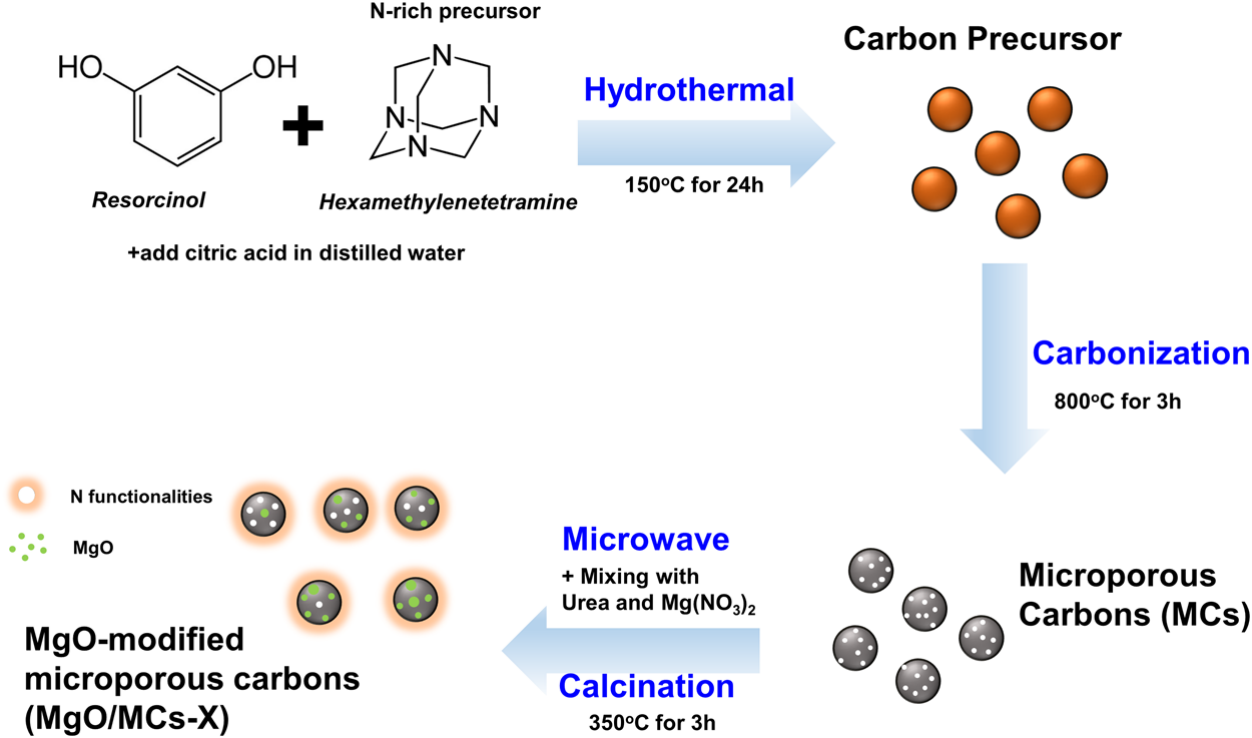
Schematic representation showing the synthetic route of MgO-modified microporous carbons (MgO/MCs-X). X refer to reaction time for microwave assisted urea-nitrate solution combustion synthesis.

In general, the accessibility of surface sites and a synergistic porous support are crucial for the adsorbent to capture the target compound efficiently from a gas stream. Two distinct strategies have been used to achieve these aims for the preparation of effective CO_2_ adsorbents based on magnesium oxide (MgO). First, the MgO can be dispersed on a porous support for enhancing CO_2_ affinity. Secondly, pores can be created in the MgO matrix itself^24–27^. In this article, we describe a green and sustainable option for the production of metal oxide-containing carbon materials. The preparation of a new type of MgO supported on spherical carbons, which were prepared by template-free hydrothermal carbonization of resorcinol resin using microwave irradiation, is described. Microwave irradiation helps to reduce the synthesis time and cost, and it can be used in industry to produce sorbents on a large scale. In addition, to maximize CO_2_ affinity to carbon adsorbents, we prepared hexamethylenetetramine with a resorcinol polymer, a nitrogen-enriched precursor, as the carbon material. In this work, carbonized materials has highly developed microporosities, denoted by microporous carbons (MCs). Figure 1 illustrates this method for the preparation of MgO-based carbon adsorbents (MgO/MCs) schematically. The CO_2_ adsorption behaviours of these materials were also investigated using simulated flue gas (15% CO_2_ in N_2_)^22, 23^. The concentration of CO_2_ in the flue gas phase was varied, and the contact time between the adsorbent and CO_2_ was limited so that intermittent adsorption was dominant over continuous adsorption. An instantaneous, rather than steady, adsorption method was therefore used to critically examine the behaviour of CO_2_ in the flue gas stream during its interaction with the MgO-based adsorbent at 313 K.

## Results and Discussion

**Figure 2:**
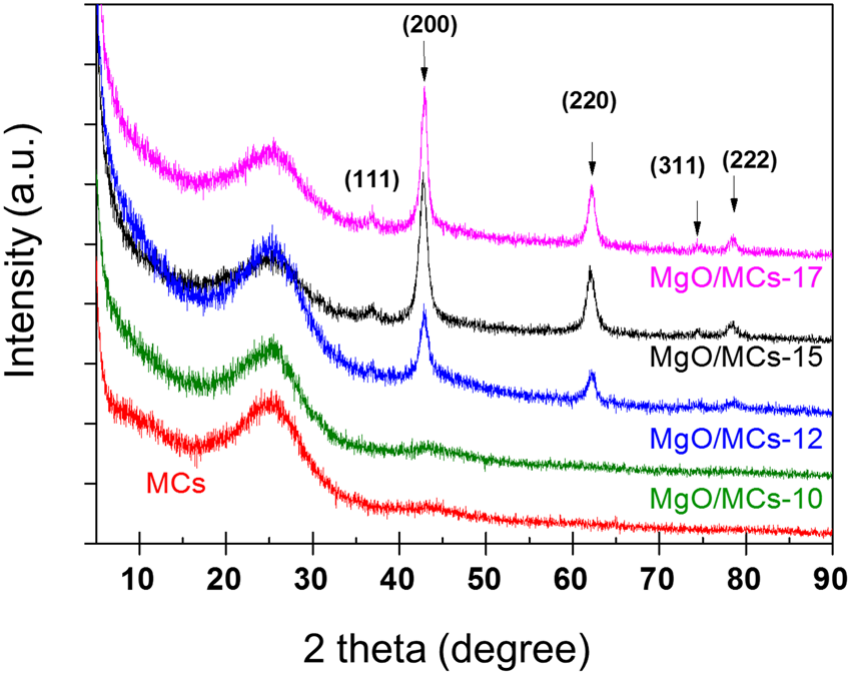
Wide-angle XRD patterns of MCs and MgO/MCs samples.

The structures and phase purities of the MgO/MCs samples were determined using X-ray diffraction (XRD) (Fig. 2). The synthesised carbon materials showed two clear peaks at about 2θ = 25° and 44°. These signals correspond to the (002) and (101) diffractions of the graphite structure, respectively. The low-intensity and wide diffraction peaks suggest that only a limited amount of graphitic material is present in the carbon materials. Additionally, strong major peaks were observed at 2θ = 43°, 62° and 79° (Fig. 2). This is a characteristic peak of MgO, which is consistent with our MgO results (Fig. S2) and the results of previous reports^24–28^. These peaks are identifiable as the (200), (220) and (222) reflections of the face-cubic centred structure of MgO. This confirms a high degree of purity and suggests that the obtained MgO/MCs samples are highly crystalline. In addition, no peaks attributed to MgO on the MCs were observed. In the XRD patterns, the intensities of the reflections derived from the MgO increase with increasing reaction time in the microwave oven; this shows that the amount of MgO in the samples is proportional to the reaction time and reaches a maximum at about 17 min. To confirm this point, an inductively coupled plasma-optical emission spectrometry (ICP-OES) analysis was performed to determine the elemental composition of the particle as a whole (including bulk and surface). The ICP-OES data (Table 1) show that the Mg content increases to approximately 15% in the MgO/MCs-17. This suggests that the MgO particles are formed on a carbon matrix as a result of oxidation of urea by the nitrate^29–31^. The increase in supplied energy with increasing reaction time enables equilibrium of the following reaction (1) to be achieved rapidly^32^.

1 $${\mathrm{2Mg}(\mathrm{NO}}_{{\rm{3}}}{)}_{{\rm{2}}}\cdot {{\rm{6H}}}_{{\rm{2}}}{\rm{O}}+{\mathrm{6CO}(\mathrm{NH}}_{{\rm{2}}}{)}_{{\rm{2}}}+{{\rm{4O}}}_{{\rm{2}}}\,\mathop{\longrightarrow }\limits^{microwave}\,{\rm{2MgO}}+{{\rm{24H}}}_{{\rm{2}}}{\rm{O}}+{{\rm{6CO}}}_{{\rm{2}}}+{{\rm{8N}}}_{{\rm{2}}}$$

**Table 1:** Textural properties and Mg loading amounts of the prepared MgO/MCs samples. Micropore volume and mesopore volume were determined using Dubinin–Radushkevich (D–R) equation and Barrett–Joyner–Halenda (BJH) model, respectively. Mg and N amounts were measured by XPS and ICP-OES analysis. CO_2_/N_2_ selectivity was calculated by Henry’s law constant using virial equation. CO_2_ uptakes were investigated at flue gas condition (313 K, 15% CO_2_/85% N_2_, 1 bar).

Specimens	S_BET_ (m^2^/g)	V_micro_ (cm^3^/g)	V_meso_ (cm^3^/g)	XPS result		ICP-OES	Selectivity (313 K)	CO_2_ uptakes (mmol/g)
				N amounts (at.%)	Mg amounts (at.%)	Mg amounts (at.%)		
MCs	760	0.315	0.016	4.76	—	—	12.6	0.73
MgO/MCs-10	325	0.143	0.114	5.97	0.46	0.24	24.4	0.80
MgO/MCs-12	228	0.107	0.132	4.63	5.19	4.60	58.8	1.22
MgO/MCs-15	89	0.056	0.105	2.55	8.64	11.85	51.6	1.01
MgO/MCs-17	13	0.007	0.062	2.36	11.48	15.06	40.3	0.69

**Figure 3:**
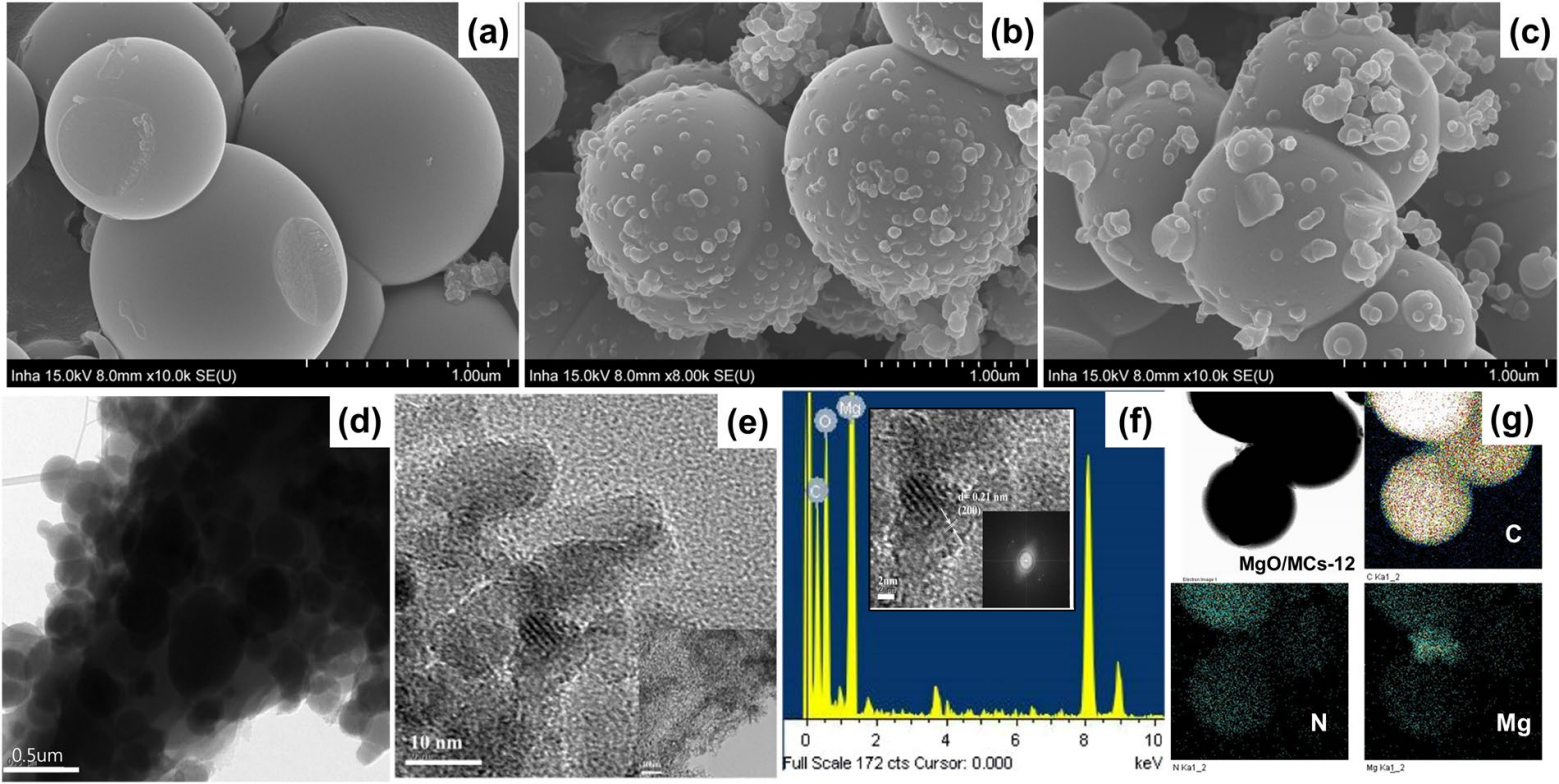
HR-SEM images of (**a**) MCs, (**b**) MgO/MCs-12, and (**c**) Mg/MCs-17. FE-TEM images of (**e**) MCs and (**f**) MgO/MCs-12, (**g**) EDS spectrum (Inset: small crystalline MgO particles) and (**h**) elemental mapping of MgO/MCs-12, C, N, and Mg, respectively.

The high-resolution scanning electron microscopy (HR-SEM) and field emission transmission electron microscopy (FE-TEM) images of the MCs spheres and MgO/MCs prepared by microwave irradiation are shown in Fig. 3. The MCs particles are almost spherical in shape and have a smooth surface (Fig. 3a and d). As the reaction time for microwave irradiation is increased, MgO particles are grown and wrapped around the supporting carbon (Fig. 3b and c). It is worth noting that the microwave-assisted synthesis of the MgO/MCs provides small crystalline MgO particles (inset of Fig. 3f), with a lattice fringe spacing (d = 0.21 nm) corresponding to the (200) plane of cubic MgO crystals, which agrees with results calculated from the XRD data using Bragg’s equation^32, 33^. In addition, MgO particles have shown porous structure itself in FE-TEM images (Fig. S3). The elemental composition of the MgO nanoparticles was determined using energy-dispersive X-ray spectroscopy (EDS), as shown in Fig. 3d. EDS is a surface-sensitive technique that mainly gives the elemental composition present up to a few layers from the surface. EDS analysis was conducted on MgO/MCs-12, and the presence of Mg, O and C was observed. The elemental mapping of Mg/MCs-12 is shown in Fig. 3(g), indicating that Mg was well dispersed on the nitrogen containing surface. It is also well matched HR-SEM images (Fig. S2) and explained MgO particles growth on the surface.

**Figure 4:**
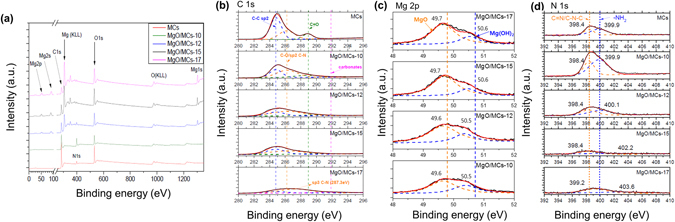
(**a**) Survey scan XPS spectra of the prepared MgO/MCs samples, high-resolution (**b**) C1s and (**c**) N1s XPS spectra of MgO/MCs samples with deconvoluted peaks.

X-ray photoelectron spectroscopy (XPS) was used to obtain information on the bound electron states on the material surfaces^34, 35^. The survey spectra were scanned in the range 50–1350 eV (Fig. 4a). Peaks at binding energies of 285 and 531 eV, corresponding to C1s and O1s, respectively, were observed for all samples. In the deconvoluted C1s XPS spectra (Fig. 4b), three clear peaks, at 284.8, 286.2 and 288.9 eV, were detected. The peaks at 284.8 eV are attributed to C–C/C–H bonds^36, 37^, and the peaks at 286.2 eV are assigned to C–N or C–O bonds^22, 36^. There was a relatively weak peak at 288.9 eV, corresponding to carbonyl carbon (C=O) bonds formed during annealing^36–38^. In addition, a peak at 291.4 eV, assigned to surface carbonates (O–C=O), was observed in the C1s XPS spectrum of MgO/MCs-17. The decrease in the peak intensity at 284.8 eV, corresponding to C1s, indicates that some of the carbon was overlapped by MgO particles or removed by oxidation at 623 K in air. Peaks related to Mg1s, Mg2s and Mg2p appeared in the XP spectra (Fig. 4a) at 1303, 89 and 51 eV, respectively; these are in agreement with the values reported for MgO^39, 40^. In particular, the increasing Mg2p intensities in the XPS spectrum confirm the presence of Mg^2^+ ions; unsaturated Mg sites, which have a strong affinity for CO_2_ at low pressure^24–26^, become increasingly packed with increasing microwave irradiation time (Fig. 4c). As observed in Fig. 4c, two peaks at binding energy at 49.7 and 50.6 eV fit the Mg 2p curve. It should be noted that the peak at the binding energy of MgO was enhanced intensity as increasing reaction time. The peak intensity of N1s at 400 eV decreased and then disappeared after 15 min of reaction time, indicating that the Mg-modified process using microwave irradiation contributed to the reduction of nitrogen components at the surface. In the high-resolution N1s spectrum (Fig. 4d), the peak with a binding energy of 399.9 eV corresponds to the amine group in primary alkyl amines (R–NH_2_), with imide and/or secondary amine contributions at 398.4 eV (C=N, C–N–C)^22, 38, 41^. The microwave treatment results in the transformation of amine groups to other types of nitrogen groups: quaternary/graphitic nitrogen (401.2 eV) and pyridine N-oxide (403.6 eV)^22, 38, 41^. These do not contribute significantly to the CO_2_ adsorption capacity, because they are less basic than amines^41^. Peaks arising from the oxidation of polycrystalline Mg (the main Mg KL23 and L23 peaks) were found in the range 304–308 eV, and no peaks associated with metallic Mg were observed^35^.

**Figure 5:**
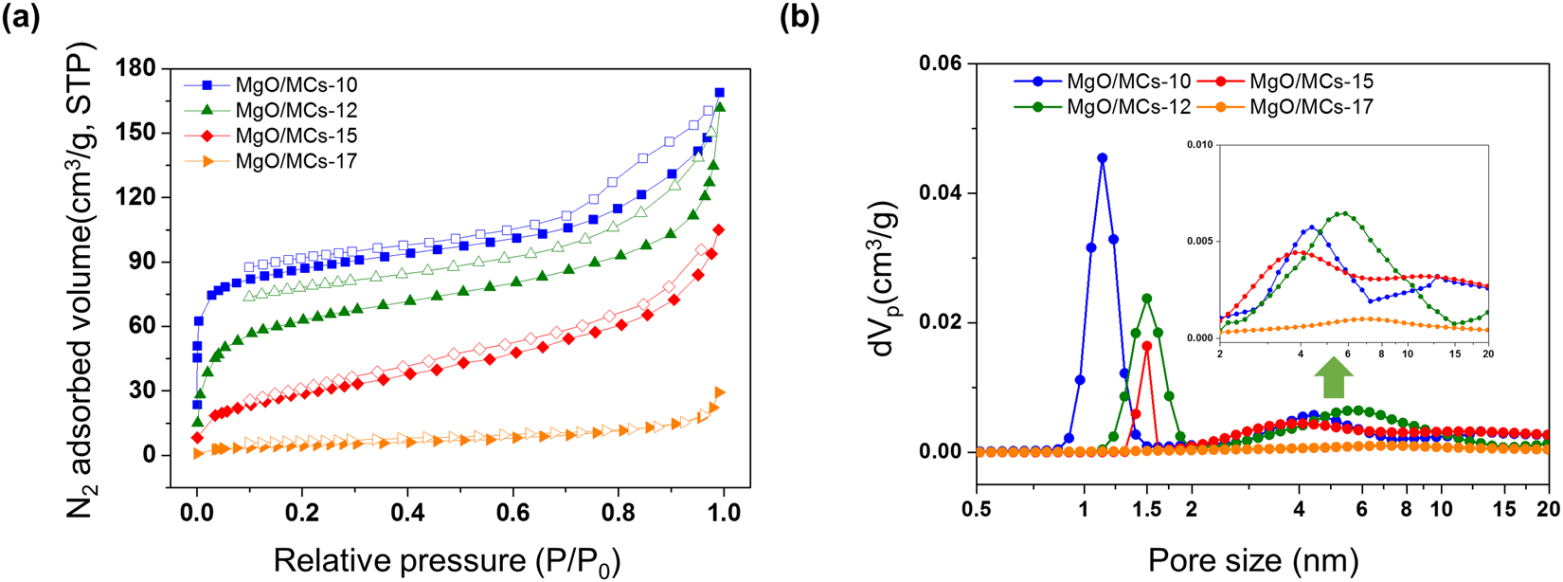
(**a**) 77 K/N_2_ adsorption (closed symbols) and desorption (open symbols) isotherms and (**b**) NLDFT pore size distributions of the prepared samples. Inset indicates mesopore size distribution.

Figures 5a and S5 show the N_2_ adsorption/desorption isotherms at 77 K of all samples. The pore size distributions determined using non-local density functional theory (NLDFT) methods are shown in Figs 5b and S5. The isotherm of the parent MCs shows typical type I behaviour (IUPAC classification), with significant adsorption below a relative pressure P/P_0_ = 0.1, implying a typical microporous structure (Fig. S5a)^23^. However, the adsorption isotherms of the MgO/MCs and MgO display type IV behaviour with low N_2_ uptakes at low pressure (Figs 5a and S5b). Table 1 lists the textural properties that are indicative of the pore structure of each sample. All the MgO-loaded samples except the MCs exhibited mesoporous structures with a maximum pore size of around 5–6 nm. A high specific surface area (S_BET_) of 760 m^2^/g and micro pore volumes (V_micro_) of 0.337 cm^3^/g were obtained for the MCs, which decreased to 89 m^2^/g and 0.056 cm^3^/g after microwave-assisted reaction (MgO/MCs-15). It is clear that the microwave-assisted reaction leads to a crucial decrease of specific surface area and microporosity. In spite of the decreasing surface area and micropore volume, mesopore volumes (V_meso_) increased with the MgO loading process (Table 1). When the microwave irradiation time was increased from 10 to 17 min, the network size increased from 1.62 to 5.6 nm, indicating that modifying was an important influence on the pore size and the final structure as a function of reaction time (Fig. 4b). It was indicated that mesopores can be developed on the carbon surface or in the MgO matrix itself (Fig. S5a). Until the reaction for 12 min, V_meso_ increased from 0.016 cm^3^/g (for MCs) to 0.135 cm^3^/g (for MgO/MCs-12) in the samples. However, V_meso_ reduced gradually over 15 min, because MgO particles blocked mesopore itself during reaction. It is also can be explained HR-SEM images, MgO Particles can have the effect of blocking pores as they grow or accumulate (Fig. S4). More detailed comparisons of the prepared samples are shown in Fig. S6, correlations between micro/meso pore volumes and Mg contents on microwave reaction times on MgO/MCs.

**Figure 6:**
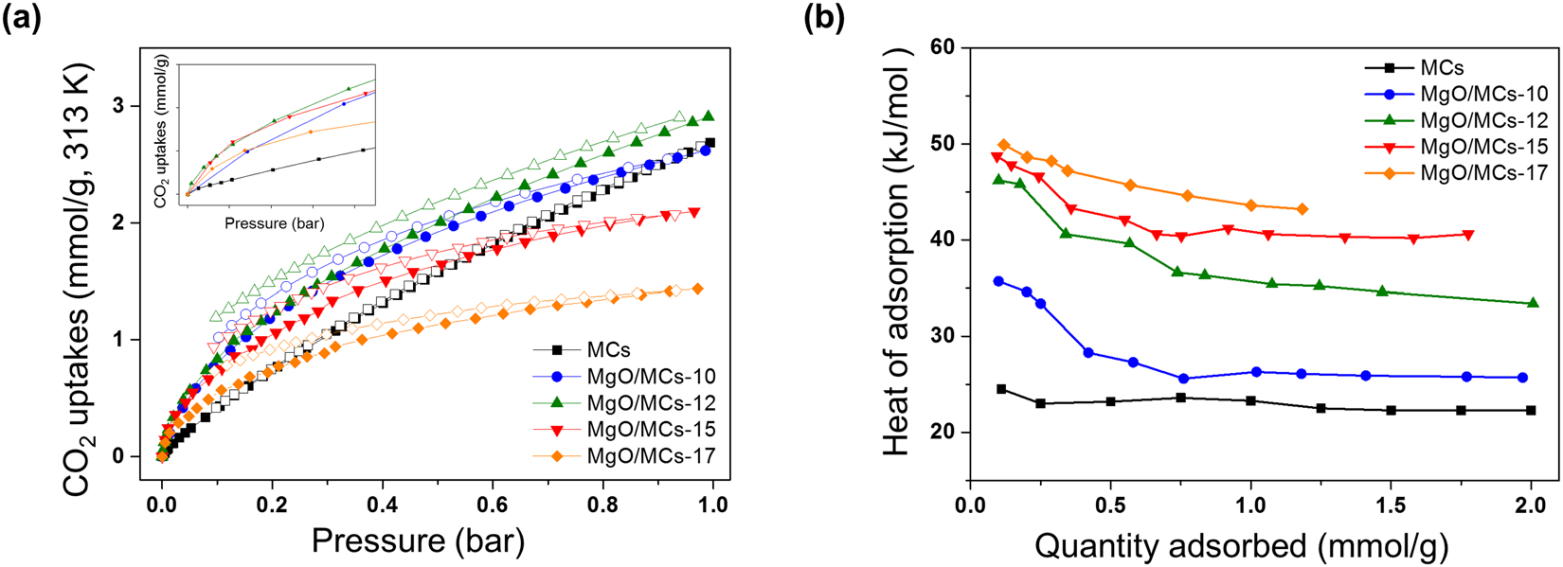
(**a**) CO_2_ adsorption/desorption isotherms of the preparation samples at 313 K. Inset indicates CO_2_ adsorption isotherms at low relative pressure (below the 0.05 bar). (**b**) Heat of adsorption on CO_2_ using Clausius–Clapeyron equation.

The CO_2_ and N_2_ adsorption/desorption isotherms of the prepared MgO/MCs samples were measured at 313 K and 1 bar using a pressure swing analysis, as presented in Figs 6a and S7. The heat of adsorption of CO_2_ can be calculated from the CO_2_ adsorption isotherms at 313 K and 323 K using the Clausius–Clapeyron equation (Fig. 6b). For CO_2_ selective adsorption over N_2_, CO_2_ affinity should be higher than N_2_ affinity. Gas affinity was determined by Henry’s law constant (*K*), which was calculated from the gas isotherm on the basis of the virial equation originally proposed for monolayer adsorption^42, 43^. Against this background, CO_2_/N_2_ selectivity (S) for gas component is CO_2_ over N_2_:

2 $${S}=\frac{{K}_{CO2}}{{K}_{N2}}$$

MgO particles tend to self-assemble during microwave irradiation, resulting in a strong reduction in the specific surface areas and micropore volumes, including nitrogen components on the surfaces (listed in Table 1). Generally, this reduction decreases the CO_2_ adsorption performance (i.e. uptake and selectivity). However, in this case, the MgO/MCs-12 attained better CO_2_ adsorption performance (2.91 mmol/g, *S*: 58.8) than the parent material (2.68 mmol/g, *S*: 12.6), because CO_2_ adsorption not only contains surface characteristics but also porosities. In addition, the initial adsorption amount in the CO_2_ adsorption isotherm is increased by introducing MgO particles (Fig. 6 inset). MCs represent the heat of adsorption values in the near 20 to 25 kJ/mol, which indicates to the physisorption of CO_2_ onto the carbon surface. This result is well matched the value of the previously reported physical adsorbents. On the other hand, the heat of adsorption values of MgO/MCs tends to increase and shows a value of about 45 kJ/mol. In the case of MgO/MCs 17, it shows a high value of more than 50 kJ/mol in the initial state, and gradually decreases to 40 kJ/mol. These results imply that MgO particles enhance CO_2_ affinity and interaction strength in range of physisorption. This may be because of the evolution of Mg components on the surfaces of the modified materials. For example, compared with MgO/MCs-10 (Mg ~0.46 at % and N ~5.97 at %), the CO_2_ uptake of MgO/MCs-12 (Mg ~5.19 at % and N ~4.63 at %) is significantly higher, although it contains half the amount of nitrogen on its surface (Fig. 4). In this case, the CO_2_-attracting ability of the metal oxide functional groups may overlap with those of nitrogen groups. This is explained by the amount of MgO loaded on the surface affecting the basic surface characteristics of the modified materials, facilitating CO_2_ adsorption, because CO_2_ is a Lewis acid^10^. In other words, some of the CO_2_-attracting ability probably involves physisorption of linearly oriented CO_2_ by ion–dipole interactions, which improves the selective CO_2_ capture of the MgO-modified samples under a mixed gas flow.

**Figure 7:**
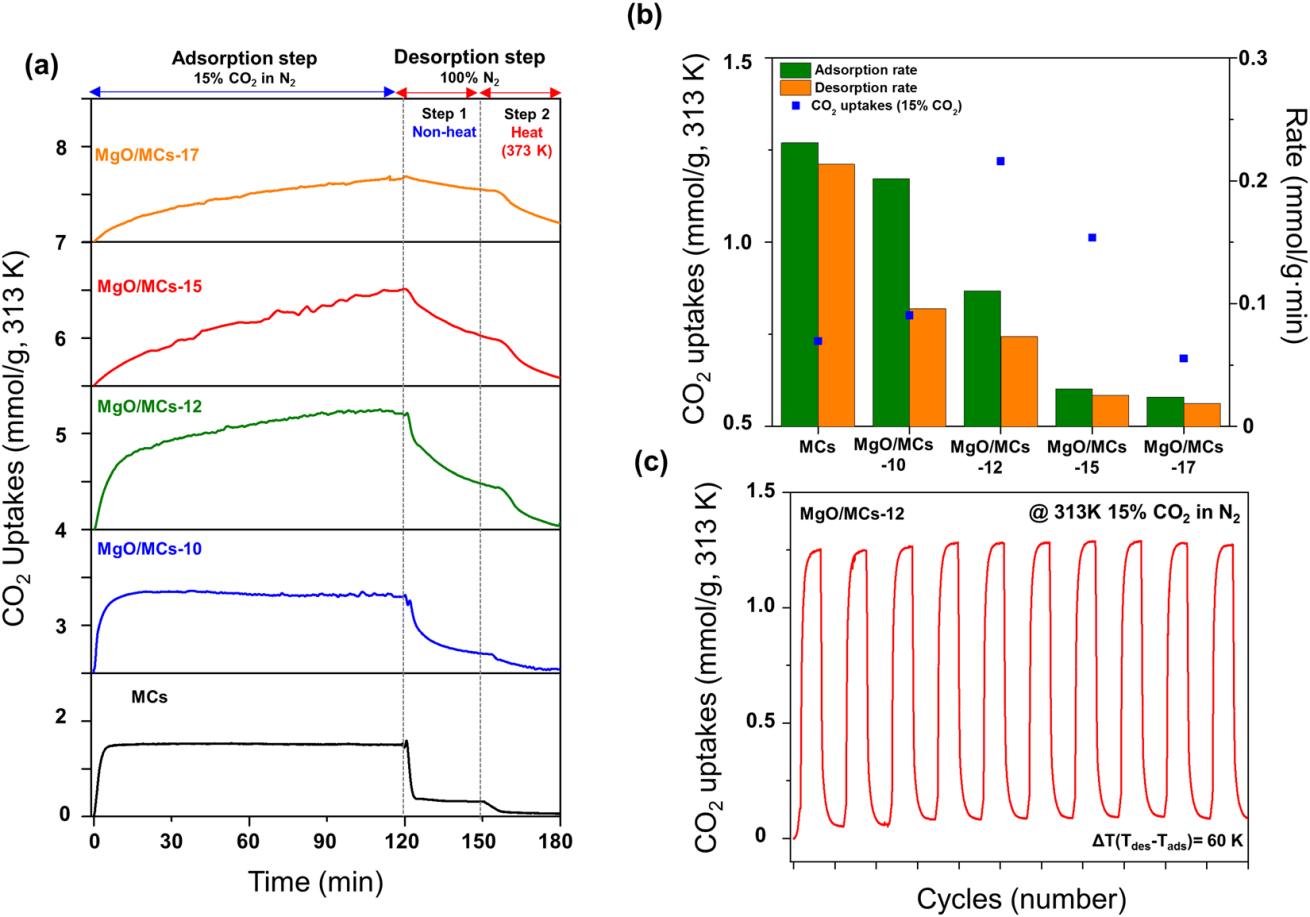
(**a**) Time-domain CO_2_ adsorption/desorption curves and (**b**) CO_2_ uptakes and adsorption/desorption rates of the preparation samples at 313 K in flue gas condition. (**c**) Time-domain CO_2_ adsorption/desorption curves of MgOMCs-12 over 10 cycles (adsorption: 15% CO_2_, 313 K 2 h desorption: 100% N_2_, 373 K for 1 h).

The multi-component CO_2_ adsorption performances were investigated by the gravimetric method^22, 23^. The multi-component adsorption method is carried out routinely and with high accuracy for validating real processes compared to the single-component adsorption method. Figure 7a shows the CO adsorption/desorption curves of the prepared samples under flue gas conditions at 1 bar. In addition, for better understanding the kinetics of CO_2_ adsorption under flue gas conditions, the adsorption/desorption rates of the samples were also obtained, which can be calculated from the first derivation of the obtained adsorption curves (Fig. S10).

In the adsorption step, the CO_2_ uptake of MCs sharply increased and was maintained constant after uptake attained saturation, which means CO_2_ molecules were physically adsorbed onto the carbon surface. The CO_2_ uptake increased with increasing MgO loading onto MCs-12 (Mg content ~5%) and then dropped at higher levels (Fig. 7a). MgO/MCs-12 exhibited the highest CO_2_ uptake of 1.22 mmol/g (5.3 wt.%) under flue gas conditions. However, MgO/MCs exhibited different types of adsorption curves, which exhibited a lower adsorption rate than pristine MCs. In particular, although MgO/MCs-17 has a higher content of MgO on its surface, it has a lower ability to capture CO_2_ than the other samples. As a result of introducing MgO particles, the amount of CO_2_ uptake is increased, but the adsorption rate is gradually decreased. The formation of increasing amounts of MgO may fill and block the pores in the pristine MCs, preventing optimum adsorption of the materials. The porosity of the materials is therefore one of several important factors that influence the CO_2_ capture performance of a sorbent. According to the comparison of MCs and MgO/MCs on CO_2_ capture, it was confirmed that adsorption rates mostly depend on microporosity compare to mesoporosity. Also, these results suggest that MgO particles not only improved CO_2_-attracting abilities for adsorption capacities but also blocked adsorption on the micropores, which reduced adsorption rates. In the desorption step, the gaseous environment was reverted to 100% N_2_ to allow desorption to take place. We proceeded in two separate steps (non-thermal and thermal steps) to test the strength of CO_2_ adsorbents’ attraction of the preparation samples. In desorption step 1, the CO_2_ uptake of MCs was found to decrease sharply after the introduction of pure N_2_. It also implied that CO_2_ was mainly physically adsorbed on the MCs. As MgO particles were introduced, desorption rates were gradually reduced due to reduced microporosity. Adsorbed CO_2_ molecules cannot be completely desorbed in non-thermal conditions, which means CO_2_ can be strongly chemisorbed on nitrogen functionalities and physisorbed on MgO particles. In desorption step 2, adsorbed CO_2_ molecules were completely desorbed at 373 K, which could be attributed to the adequate desorption energy. Adsorbed CO_2_ molecules of MgO/MCs-17 cannot be completely desorbed at 373 K for 30 min, which might be due to the lack of desorption energy causing incomplete CO_2_ desorption from the surface of MgO/MCs-17. The reusability and reproducibility of the MgO/MCs-12 were examined by performing consecutive CO_2_ adsorption/desorption runs at 313 K under flue gas conditions (Fig. 7c). CO_2_ is desorbed from the MgO/MCs-12 at 373 K in 1 h, and there is no significant change in activity after 10 cycles. There is therefore the potential for energy saving during sorbent recovery. In addition, to examine the CO_2_ adsorption uptakes by competing adsorbents, the CO_2_ uptake capacities of MgO/MCs and other adsorbent materials reported in the literature were compared^44–48^. More detailed comparisons are shown in Table S3. The improvement in the CO_2_ capture performances of MgO/MCs compared with that of the parent material is partly the result of the presence of metal oxides in the MCs structures.

## Conclusions

In this study, carbon materials containing MgO were successfully synthesised using a simple process involving carbonization and microwave-assisted reaction. We have outlined the advantages of the overall procedure from the perspectives of net energy use, synthesis time and simplicity. Through the introduction of MgO particles, it was confirmed that CO_2_ selectivity and CO_2_ uptakes were improved in the adsorbent by improving the ion-dipole interaction between the MgO and CO_2_. In addition, this work shows that not only surface characteristics but also the porosities of the MgO-modified sorbents affect the CO_2_ adsorption behaviours. The textural properties and surface chemistry both influenced the CO_2_ capture performance of the prepared carbon adsorbents. The results show that the reaction time for MgO loading influenced not only the Mg contents in the final products but also their structures. This suggests that reasonable dispersion of the MgO particles into the carbon materials effectively increases the affinity to CO_2_ molecules. It is expected that these properties of the materials will lead to improved adsorptive performance. As such, MgO/MCs have potential applications in post-combustion CO_2_ capture.

## Methods

### Materials and sample preparation

All chemicals, such as resorcinol (≥99.0%), hexamethylenetetramine (ACS reagent, ≥99.0%), citric acid (ACS reagent, ≥99.5%) and Mg(NO_3_)_2_·6H_2_O, were obtained from Sigma-Aldrich and used as received without further purification. In a typical synthesis, resorcinol (15 mmol) and hexamethylenetetramine (15 mmol) were dissolved in water (20 mL) followed by the addition of citric acid (10 mmol). The mixed solution was added to a stainless steel autoclave of volume 50 mL. The autoclave was sealed and heated in an oven at 353 K for 24 h. The precipitates were collected by filtration and washed with distilled water and ethanol. All products were dried at 353 K for 12 h. Carbonization was carried out in a tubular furnace under an inert atmosphere (N_2_ flow) at 1073 K for 3 h. During this process, a heating rate of 2 K/min was maintained. In the next step, the carbonization products, denoted by MCs, were modified with Mg(NO_3_)_2_ salt and urea (CH_4_N_2_O). Mg(NO_3_)_2_·6H_2_O is used as a oxidizer and urea used as a fuel for microwave-assisted urea-nitrate solution combustion synthesis. The stoichiometric compositions of the solution components (oxidizer and fuel) were calculated for 1 mole of Mg(NO_3_), 1.67 mol of urea required. Mg(NO_3_)_2_·6H_2_O (1.53 g) and urea (0.6 g) were dissolved in distilled water and stirred for 15 min to obtain a clear solution, and then the MCs material (0.5 g) was added. The mixture was stirred continuously for 2 h and then placed in a domestic microwave oven (2.45 Hz, 750 W) for 10–17 min. The resulting materials were removed by filtration, washed with distilled water and ethanol solution and dried at 333 K in an oven overnight. The last step in the preparation was calcination for 3 h at 623 K. The final products are denoted by MgO/MCs-x, where x represents the reaction time with metal salts under microwave radiation.

### Characterization

Before characterizations and measurements, all samples were degassed 120 °C in oven for 12 h. The crystal structures of the synthesised samples were determined using powder XRD (D2 PHASER, Bruker, Germany) with Cu-Kα radiation at 30 kV and 10 mA (λ = 1.5406 Å). ICP-OES (Optima 7300DV, Perkin Elmer, USA) was used to determine the elemental contents of the prepared samples. XPS was used to investigate the elements on the sample surfaces. The XPS spectra were obtained using a monochromatic Al-Kα X-ray source (Thermo Scientific K-Alpha photoelectron spectrometer, Thermo Fisher Scientific Inc.). The surface morphologies of the MCs were examined using HR-SEM (SU8010, Hitachi co.) and FE-TEM (JEM2100F, JEOL Co.). The prepared samples for HR-SEM were sputter-coated with platinum before measurement. The textural properties of the MCs were investigated using a gas adsorption analyser (Belsorp-max, BEL Co.) in the pressure range 0–1 bar. Isotherms were measured at 77 K in liquid nitrogen baths. The specific surface areas and micropore volumes were determined using the Brunauer–Emmett–Teller (BET) equation and Dubinin–Radushkevich (D–R) equation. The mesopore volumes were determined using the Barrett–Joyner–Halenda (BJH) model. The pore size distributions were investigated by the NLDFT method. The CO_2_ and N_2_ adsorption–desorption isotherms at 313 K for the series of MgO/MCs were also measured to investigate CO_2_ adsorption using a gas adsorption analyser (Belsorp-max, BEL Co.), as mentioned above.

### CO_2_ adsorption–desorption cycle experiments under flue gas conditions

CO_2_ adsorption–desorption cycle experiments under flue gas conditions were carried out and analysed using the thermal gravimetric analyser (TGA, Perkin Elmer, USA) as follows: 5 mg of sample was loaded in a platinum pan and heated to 200 °C at a rate of 5 °C/min. This temperature was maintained for 300 min to degas the sample, and then the system was cooled to 313 K (5 °C/min) under 100% N_2_ gas. In the adsorption process, the gas flow composition was changed, and the system was maintained at 313 K for 120 min under a gas composition of 15% CO_2_ in N_2_ as flue gas in fossil fuel power plants. There were two desorption steps, non-thermal and thermal steps, as follows: (1) 15% CO_2_ gas flow was changed to 100% N_2_ gas and maintained for 30 min in the non-thermal desorption process. (2) The system was maintained at 100% N_2_ gas conditions and heated to 373 K for perfect degassing.

## Electronic supplementary material

Supplementary Info
