# Supplementary material for: Facile Synthesis of MgO-Modified Carbon Adsorbents with Microwave- Assisted Methods: Effect of MgO Particles and Porosities on CO2 Capture
Source: Sci Rep. 2017 Jul 18;7:5653. doi: 10.1038/s41598-017-06091-5 (PMC5515889; doi:10.1038/s41598-017-06091-5)
Supplement: Supplementary file 1 — Supplementary Info [file 41598_2017_6091_MOESM1_ESM.pdf]

Supporting Information

Facile Synthesis of MgO-Modified Carbon Adsorbents with Microwave-Assisted Methods : Effect of MgO particles and Porosity on CO_2 Capture

Young-Jung Heo and Soo-Jin Park*

Department of Chemistry, Inha University, 100 Inharo, Incheon 22212, Korea

* Corresponding authors. Tel.: +82-32-876-7234; Fax: +82-32-867-5604.

E-mail address: sjpark@inha.ac.kr (S. -J. Park)

Calculation of stoichiometric composition on solution combustion synthesis

We used solution combustion synthesis, which is an effective, low cost, simplicity in preparation of the metal oxide. So, urea used as a fuel in microwave assisted urea-nitrate solution combustion synthesis. $Mg(NO_3)_2 \cdot 6H_2O$ is used as a oxidizer and urea (CH_4N_2O) used as a fuel. The stoichiometric compositions of the solution components (oxidizer and fuel) were calculated as follow;

Oxidizing valency of $Mg(NO_3)_2$: Mg= +2, 2N= 0, 6O=-12, Total= -10

Reducing valency of CH_4N_2O : C=+4, 4H=+4, 2N =0, O=-2 Total= +6

For 1 mole of $Mg(NO_3)$, 1.67 mol of urea required.

Calculation of Henry's law selectivity

To obtain reliable Henry's law constants, a virial-type expression comprising the temperature-independent parameters a_i and b_i was applied;

$$\ln P = \ln N + \frac{1}{T}\sum_{i=0}^{m} a_i N^i + \sum_{i=0}^{n} b_i N^i$$

Where *P*, *N*, and *T* are the pressure, adsorbed amount, and temperature, respectively. a_i and b_i are virial coefficients, which are the temperature independent fitting parameters. *m* and *n* represent the number of coefficients required to adequately describe the isotherms. The fitting of all isotherms in this work have R^2 greater than 0.999. The Henry's law constant (*K*) at the temperature *T* can be calculated;

$$K = \exp(-b_0) \cdot \exp(-a_0 / T)$$

The Henry's law selectivity for pure gas component *i* over *j* is given as;

$$S_{ij} = K_i / K_j$$

The Henry's law constants and fitting parameters for are listed in Table S1.

Calculation of heat of adsorption (ΔH_{ads})

The heat of adsorption (ΔH_{ads}) was calculated using the adsorption data collected at 313 and 323 K and the Clausius-Clapeyron equation.

$$\Delta H_{ads} = -R\left[\frac{d \ln P}{d(1/T)}\right]$$

where R is the gas constant, and P is the equilibrium partial pressures of CO_2 adsorbed at given temperature T.

Supplementary figures

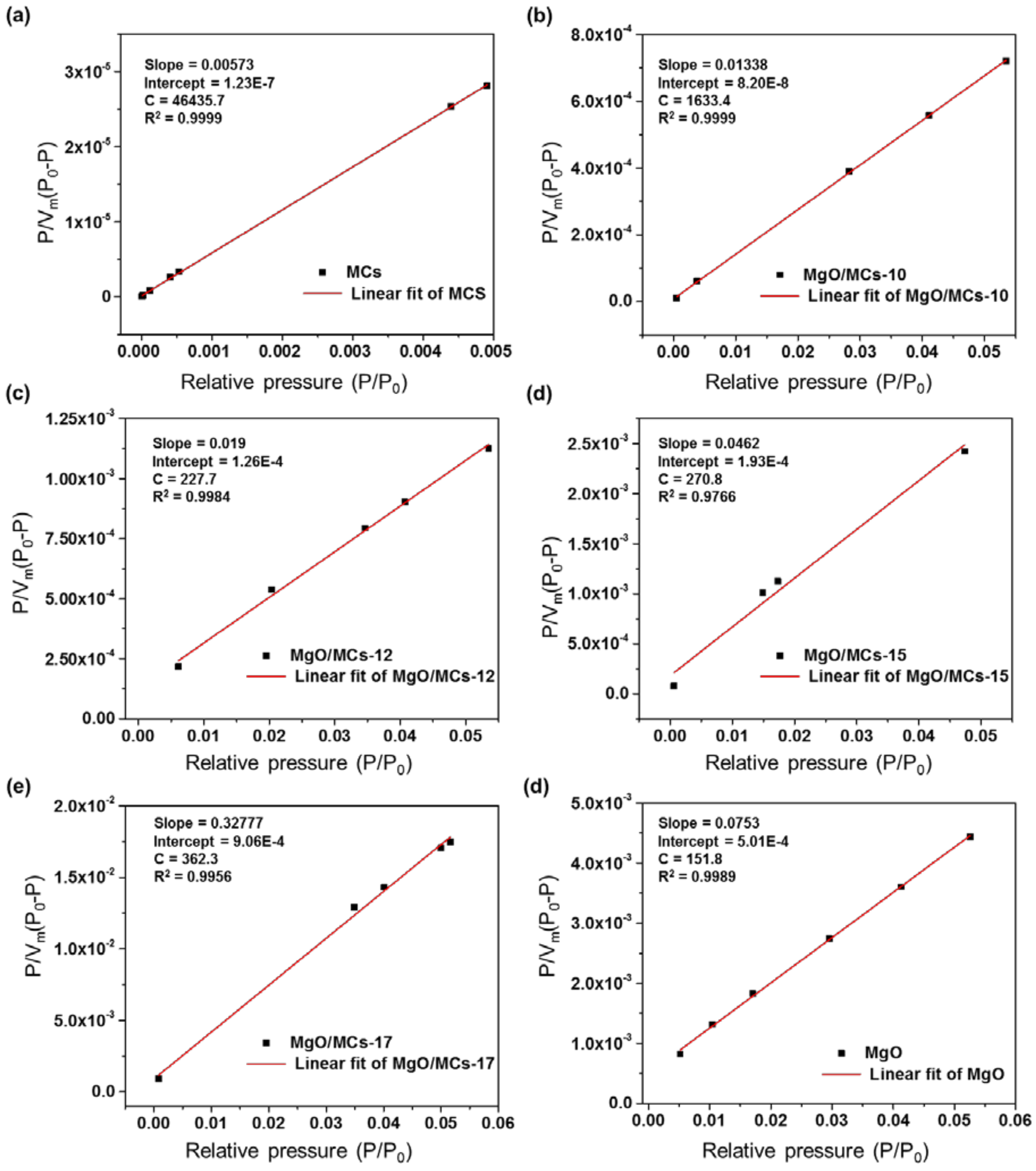

Figure S1. BET plots of the MgO/MCs samples from their corresponding 77K/N_2 adsorption isotherms.

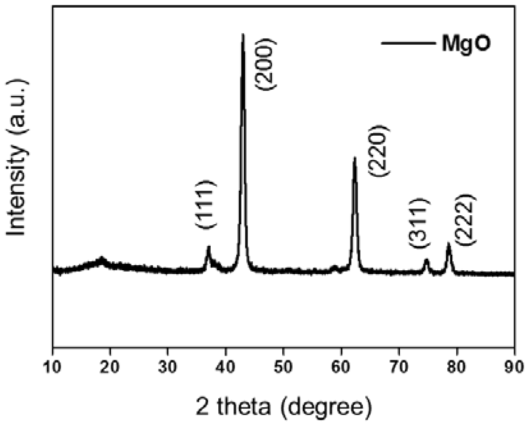

Figure S2. XRD patterns of MgO nanoparticles.

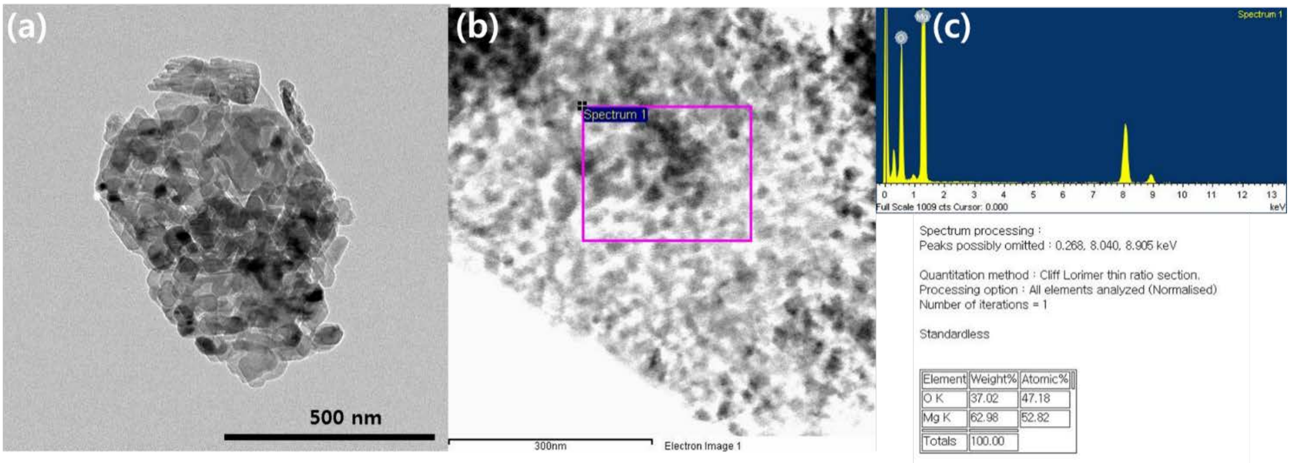

Figure S3. (a,b) FE-TEM images and (c) EDS spectrum of MgO nanoparticles.

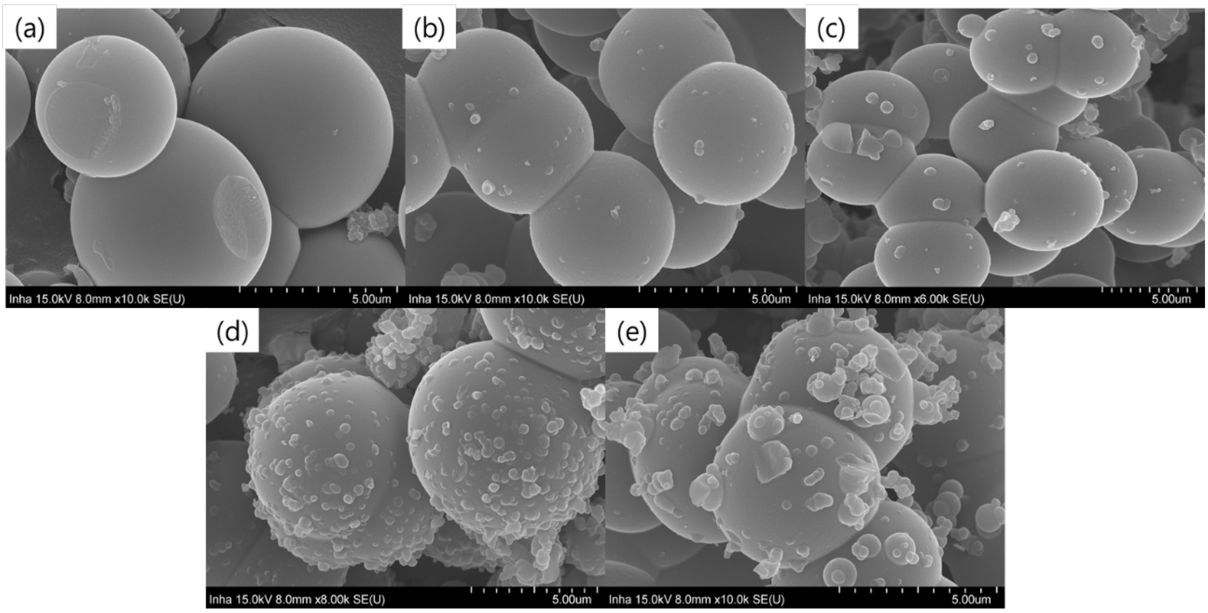

Figure S4. HR-SEM images of (a) MCs, (b) MgO/MCs-10, (c) MgO/MCs-12, (d) MgO/MCs-15 and (e) MgO/MCs-17.

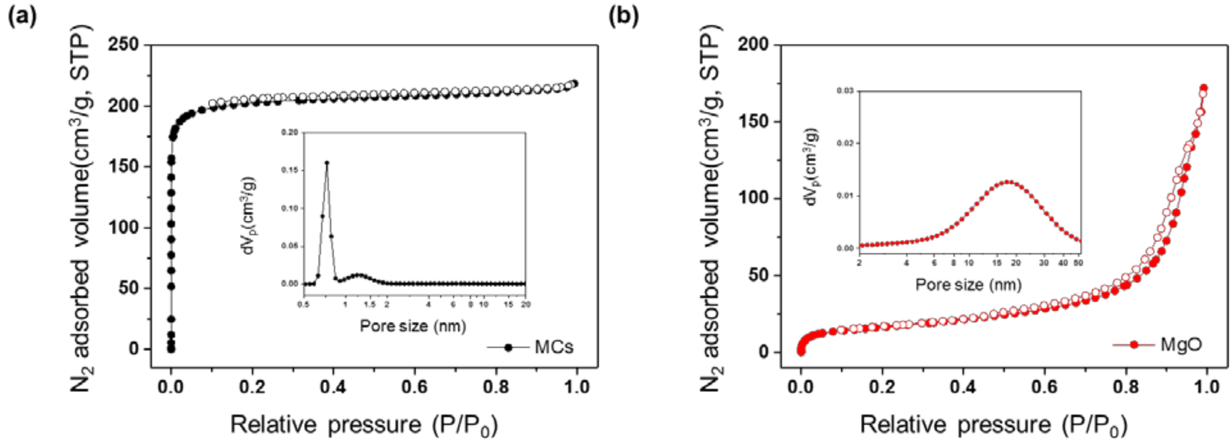

Figure S5. 77 K/N_2 adsorption (closed symbols) and desorption (open symbols) isotherms of the (a) MCs and (b) MgO nanoparticles. Inset indicates NLDFT pore size distribution.

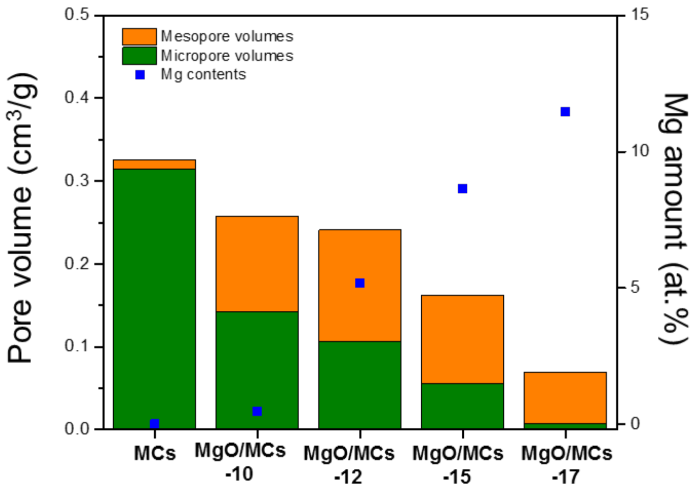

Figure S6. Correlations between micro/meso pore volumes and Mg contents on microwave reaction times on MgO/MCs.

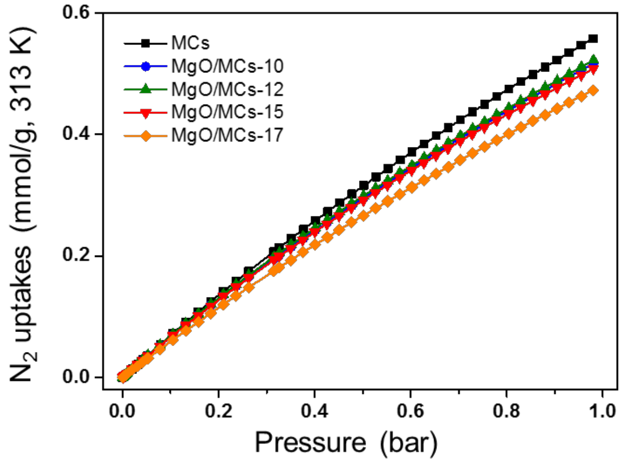

Figure S7. N_2 adsorption isotherms of the preparation samples at 313 K.

Table S1. Henry's law constant (*K*), virial fitting results and selectivity of MgO/MCs samples.

Specimens	K_{CO2} (mmol g^{-1} kPa^{-1})	K_{N2} (mmol g^{-1} kPa^{-1})	CO_2 fitting R^2	N_2 fitting R^2	Selectivity
MCs	0.0857	0.00681	0.9999	0.9999	12.6
MgO/MCs-10	0.1672	0.00686	0.9997	0.9999	24.4
MgO/MCs-12	0.4095	0.00697	0.9998	0.9997	58.8
MgO/MCs-15	0.3511	0.00680	0.9999	0.9996	51.6
MgO/MCs-17	0.2388	0.0592	>0.9999	0.9998	40.3

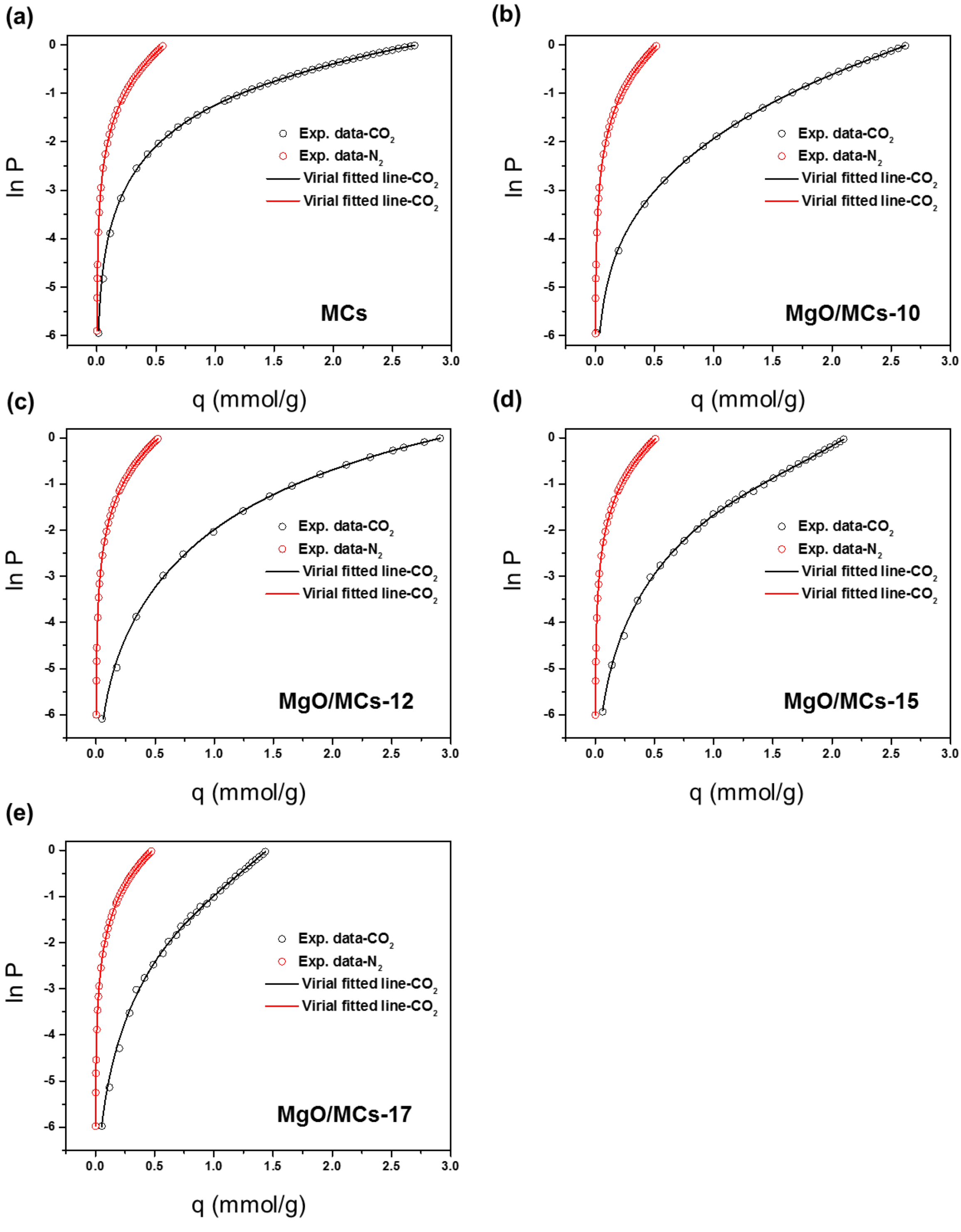

Figure S8. Virial fitting results for CO_2 and N_2 adsorption of the samples at 313 K.

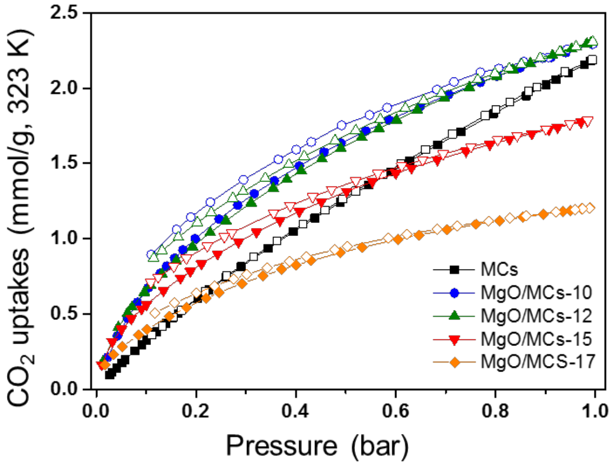

Figure S9. CO_2 adsorption/desorption isotherms of the samples at 323 K.

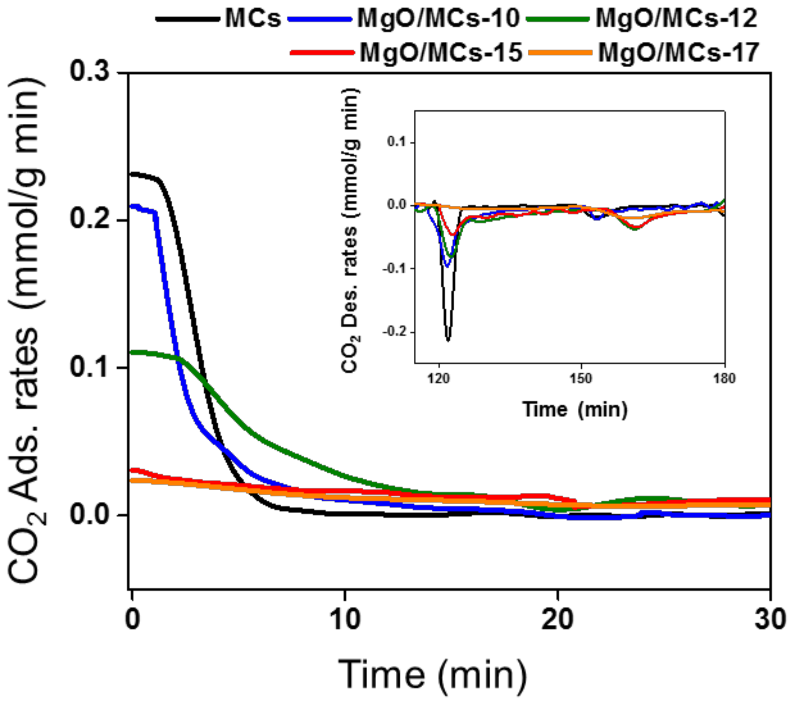

Figure S10. Adsorption/desorption rates of the samples were also obtained, which can be calculated from the first derivation of the obtained adsorption curves.

Table S2. CO_2 capturing performances of (i.e. CO_2 uptakes, adsorption rates and CO_2 uptake retention) of the samples evaluated under flue gas condition (15% CO_2 /85% N_2) at various temperatures.

Specimens		313 K
MCs	CO_2 uptakes[a] (mmol g^{-1})	0.73
	Adsorption rates[b] (mmol g^{-1} min^{-1})	0.23
MgO/MCs-10	CO_2 uptakes[a] (mmol g^{-1})	0.80
	Adsorption rates[b] (mmol g^{-1} min^{-1})	0.20
MgO/MCs-12	CO_2 uptakes[a] (mmol g^{-1})	1.22
	Adsorption rates[b] (mmol g^{-1} min^{-1})	0.11
MgO/MCs-15	CO_2 uptakes[a] (mmol g^{-1})	1.01
	Adsorption rates[b] (mmol g^{-1} min^{-1})	0.03
MgO/MCs-17	CO_2 uptakes[a] (mmol g^{-1})	0.69
	Adsorption rates[b] (mmol g^{-1} min^{-1})	0.02

[a] CO_2 uptakes were investigated for 120 min under flue gas condition at various temperatures.

[b] Adsorption rates were determined from the highest value of the first derivation of CO_2. adsorption curves.

Table S3. Comparison of the performance of CO_2 adsorbents materials.

Specimens	Conditions	CO_2 uptakes (mmol/g)	Selectivity	Cycle	Ref.
PEI-loaded MCM-41	15% CO_2 /85% N_2 , 313 K	0.568	-	X	[44]
N-doped carbons	15% CO_2 /85% N_2 , 313 K	1.002	50	O	[22]
Mesorporous MgO	15% CO_2 /85% N_2 , 313 K	1.295	-	X	[45]
Zeolite-Y-EDA	15% CO_2 /85% N_2 , 313 K	1.4-1.1	-	O	[46]
Coppersilicate	15% CO_2 /85% N_2 , 313 K	1.3-1.4	3515	O	[47]
Titanosilicate	15% CO_2 /85% N_2 , 313 K	1.3-1.5	879	O	[48]
MgO/MCs	15% CO_2 /85% N_2 , 313 K	1.221	59	O	This work
